# Adsorptive removal of heavy metals from wastewater using Cobalt-diphenylamine (Co-DPA) complex

**DOI:** 10.1186/s13065-024-01128-z

**Published:** 2024-01-29

**Authors:** Mesfin Yimer, Shagufi Naz Ansari, Biniyam Abdu Berehe, Krishna Kanthi Gudimella, Gangaraju Gedda, Wubshet Mekonnen Girma, Nazim Hasan, Shadma Tasneem

**Affiliations:** 1 Department of Chemistry, College of Natural Science, Wollo University, P.O. Box:1145, Dessie, Ethiopia; 2 Department of Chemistry, School of Engineering, Presidency University, Bangalore, Karnataka 560064 India; 3 Department of Chemistry, School of Science, GITAM (Deemed to Be University), Rudraram, Telangana 502329 India; 4 Central Research Laboratory, K S Hegde Medical Academy, NITTE (Deemed to Be University), Deralakatte, Mangaluru, Karnataka 575018 India; 5 Department of Animal Science & Technology and BET Research Institute, Chung-Ang University, Anseong, Gyeonggi-do 17546 Republic of Korea; 6 Department of Chemistry, College of Science, Jazan University, P.O. Box 114, Jazan, 45142 Kingdom of Saudi Arabia

**Keywords:** Wastewater, Adsorption, Cu-DPA, Real sample, Freundlich isotherm

## Abstract

Heavy metals like Cadmium, Lead, and Chromium are the pollutants emitted into the environment through industrial development. In this work, a new diphenylamine coordinated cobalt complex (Co-DPA) has been synthesized and tested for its efficiency in removing heavy metals from wastewater, and its adsorption capacity was investigated. The effectiveness of heavy metals removal by Co-DPA was evaluated by adjusting the adsorption parameters, such as adsorbent dose, pH, initial metals concentration, and adsorption period. Heavy metal concentrations in real sample were 0.267, 0.075, and 0.125 mg/L for Cd^2+^, Pb^2+^, and Cr^3+^ before using as-synthesized Co-DPA to treat wastewater. After being treated with synthesized Co-DPA the concentration of heavy metals was reduced to 0.0129, 0.00028, 0.00054 mg/L for Cd^2+^, Pb^2+^, and Cr^3+^, respectively, in 80 min. The removal efficiency was 95.6%, 99.5%, and 99.5% for the respective metals. The adsorption process fitted satisfactorily with Freundlich isotherm with R^2^(0.999, 0.997, 0.995) for Cd^2+^, Pb^2+^, and Cr^3+^, respectively. The kinetic data obeyed the pseudo-second order for Cd^2+^ and Cr^2+^ and the pseudo-first order for Pb^2+^. Based on the results obtained within the framework of this study, it is concluded that the as-synthesized Co-DPA is a good adsorbent to eliminate heavy metal ions like Cd^2+^, Pb^2+^, and Cr^3+^from wastewater solution. In general, Co-DPA is a promising new material for the removal of heavy metal ions from water.

**Graphical Abstract:**

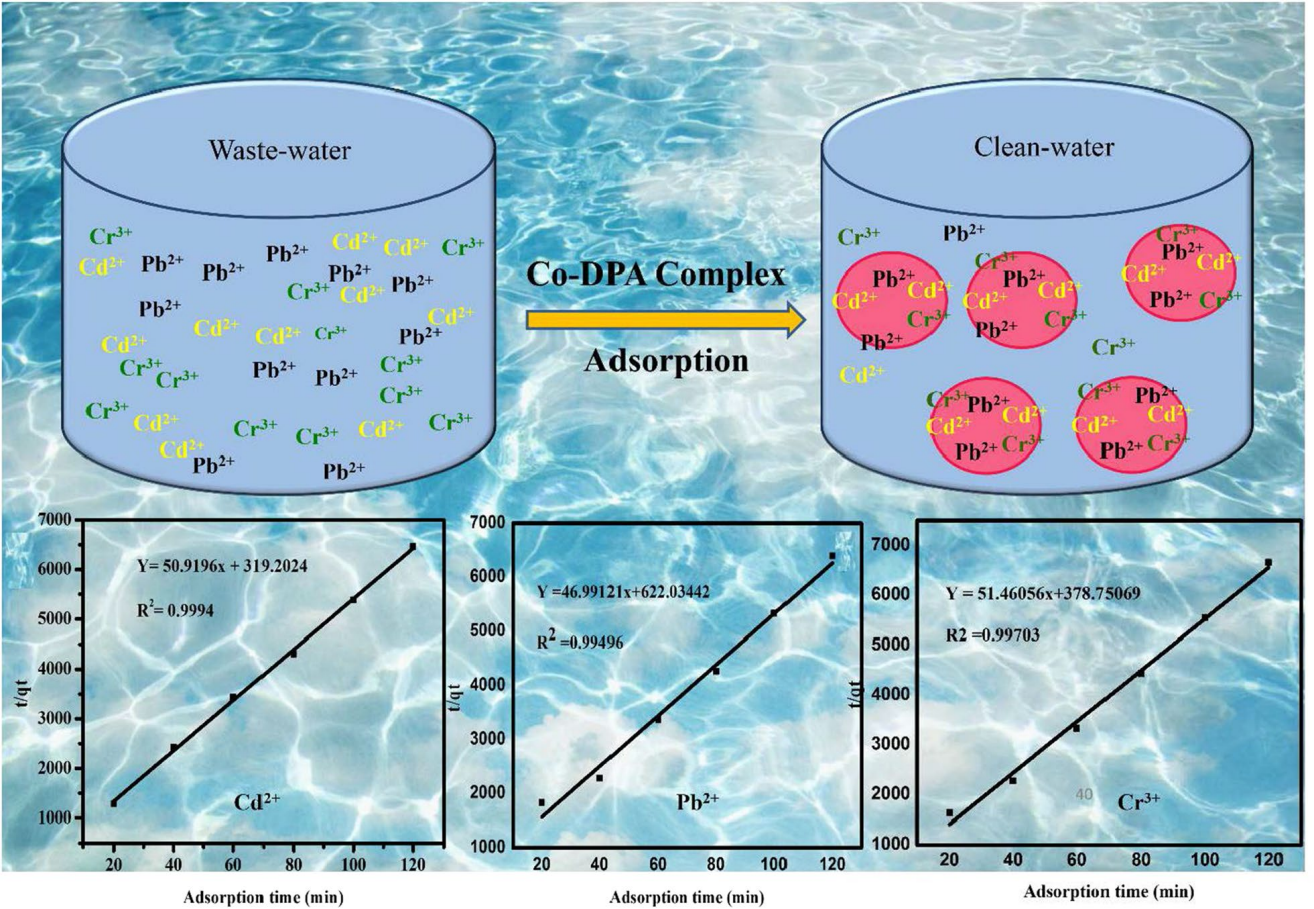

## Introduction

Heavy metal ions are one of the major sources of water contamination [1]. Exposure to heavy metal ions consequences in a huge threat to both human health and ecological balance due to their carcinogenic nature [1–3]. Wastewater originated from industries such as paint manufacture, mining activities, metal plating, electroplating industry, batteries, pesticides, and fluidized bed bioreactors, etc., are known to have high concentrations of various heavy metal ions, such as copper (Cu), arsenic (As), cadmium (Cd), mercury (Hg), and lead (Pb) [1–3]. This heavy metal-polluted wastewater is discharged into the natural water resources, threatening human health and the ecosystem [2–5]. The heavy metals are carcinogenic and non-biodegradable, which get piled up in living organisms, further generating several critical health issues and syndromes such as fleeting growth and development, impairment of the nervous system, obstructive lung disease, and death in extreme cases [5–7]. Lead and cadmium both have nephrotoxic effects and are linked to bone deterioration and encephalopathy at high exposure levels [8–12]. Hence, industrial wastewater must be treated before releasing into the rivers or ponds to exclude these toxic heavy metals. The World Health Organization has set guidelines for the acceptable levels of lead and cadmium in wastewater, which are 0.015 mg/L and 0.01 mg/L, respectively [8, 11, 12]. Hexavalent chromium is poisonous and carcinogenic at high enough quantities resulting in Ulcers and perforation of nasal septum, and respiratory cancer [7].

Various conventional methods have been investigated for the removal of heavy metal ions like precipitation, ion exchange, advanced oxidation processes, membrane filtration, electrocoagulation (EC), reverse osmosis, etc. [13–20]. However, most of these techniques have substantial operating costs and require the disposal of the solid waste they produce. The adsorption method is a superior technique due to its lesser operating cost, straightforward implementation, high removal efficiency, easy regeneration of the adsorbent, and production of little or no byproduct [21–25]. The mechanism of the adsorption process is based on the physicochemical properties of heavy metals and adsorbent and operating conditions (i.e., pH value, adsorbent amount, temperature, adsorption time, and initial concentration of metal ions) [26–28]. Usually, heavy metal ions can be adsorbed on the surface of the adsorbent via intermolecular interactions. The amputation of heavy metals from numerous sources of wastewater has reportedly been accomplished using commercial activated carbon [15, 29, 30], zeolites, and biomass-derived adsorbents like peanut shells [31–33], banana peel [34, 35], dry tree leaves [36], rice husk [37], tea and coffee waste [38], coconut shell powder [39], papaya seed [40], and eggshell [41]. Nevertheless, some problems, including the need for a prolonged reaction time, excessive calcination temperature, inadequate stability, short reusability, a low surface area, etc. restrict most of the available adsorbents [15, 16, 29, 31, 42]. As a result, it is crucial to create low-cost adsorbents with a large surface area utilizing straightforward synthesis techniques. The characteristics of a promising ion adsorbent are high adsorption kinetics, substantial adsorption capacities even at low contamination levels, selectivity for the target ion, stability in the aqueous environment, scalability, recoverability, and cost [25, 27, 30, 34, 36, 43–48]. Physical adsorption and chemical adsorption are the two main categories of adsorption processes [15]. Weak bonds (van der Waals force and dipolar forces) and low heats of adsorption are characteristics of physisorption [15]. The attraction between permanent dipoles and induced dipoles also allows adsorbate molecules to freely cover the whole surface of the adsorbent without changing the species' atomic or molecular orbitals. As a result, adsorbate molecules are not constrained to locations. However, chemisorption comprises the interchange of electrons between the adsorbate and the adsorbent [42]. For the elimination of Co (II) ions from aqueous solutions, activated carbons made from hazelnut shell were utilized as adsorbents displayed adsorption capacity as 58.27 mg g^−1^ [49, 50]. Activated carbon from *Borassus aethiopum* seed shells (BASS) and *Cocos nucifera* (CONS) was studied by Mohammed and Azim for the removal of Pb^2+^ and Cd^2+^ (shells) showing 99% and 95% removal for Pb^2+^ and Cd^2+^ respectively [51]. In 2020 Wang and coworkers synthesized Ferrous phosphate to treat wastewater containing EDTA-Pb from 50 mg Pb/L to < 1 mg Pb/L [52]. In 2021 Asma et al. reported sodium alginate grafted by diphenylamine as adsorbent for the removal of Co^2+^ ions through adsorption process where the adsorption capacity was dependent on the pH value [53]. The maximum removal capacity was credited to the electrostatic attraction between the metal ions and the negative charge of adsorbent surface. In 2020 Ngoc T. et al. reported hydrogen-bonded supramolecular complex for efficient removal of trace heavy metal ions from water exhibiting adsorption kinetics, up to 50 times faster than state-of-the-art materials for selective copper ion capture from water [54]. In 2018, Xu et al. reported selective adsorption of Cu (II) and SO_4_^2−^ ions through silica gel-immobilized Schiff base derivative [55]. In 2020 Jumina et al. reported the removal of Pb^2+^, Cr^2+^, and Cu^2+^ metal ions through pH Dependent adsorption process using *C*-Phenylcalix pyrogallolarene [4]. The phenolic group of the adsorbent increases the metal uptake efficiency through the ion exchange process [56]. Some recent examples of adsorbents are summarized below showing operation conditions and removal efficiency (Table 1) as comparison with our study.

**Table 1:** A comparative table to include operation conditions, and removal efficiency

S/N	Type of adsorbent	Time	Metal concentration	Amount of adsorbent	Removal efficiency	References
1	{[Cd_1.5_(btc)(bibp)·2H_2_O] H_2_ O}_n_	120 min	1000 ppm	10 mg	Pb(II) (53.9%) and Cr(VI) (73.8%)	[57]
2	α-cellulose/chitosan	60 min	60 ppm	1.0 g	Cr(II) (56%), Pb(II) (85%) and Cd (94%)	[58]
3	Non-cytotoxic and magnetic cobalt ferrite nanoparticles	30 min	450 ppm	0.1 g	Pb(II) (82%)	[59]
4	Magnetic activated carbon–cobalt nanoparticles	30 min	500 ppm	0.1 g	Pb(II) (90.6%)	[60]
5	Faujasite zeolite decorated with cobalt ferrite nanoparticles	12 h	140 ppm	1 g	Pb(II) (98.4%)	[61]
6	Multifunctional cobalt oxide nanocomposites	140 min	350 ppm	2.8 g	Pb(II) (86.89%) and Cd(II) (82.06%)	[62]
7	Polypyrrole functionalized, Cobalt oxide Graphene	120 min	400 ppm	0.01 g	Pb(II) (93.08%) and Cd(II) (95.28%)	[63]
8	Cobalt ferrite nanoparticles	60 min	400 ppm	0.1 g	Pb (96%)	[64]
9	Organosulphur-modified biochar	300 min	50 ppm	100 mg	Cd(II) (88%)	[65]
10	Activated carbon cobalt composite	20 min	450 ppm	0.1 g	Pb(II) (85%)	[60]
11	Cobalt ferrite nanoparticles	30 min	150 ppm	10 g	Cr(IV) (50.9%)	[66]
12	Co-DPA	80 min	60 ppm	3 g	Cr(II) (99.5%), Pb(II) (99.5%) and Cd (95.6%)	This study

This study aims to investigate the removal of heavy metals from wastewater using cobalt-complex (Co-DPA) under different experimental conditions. The Co-DPA was prepared from the cobalt nitrate hexahydrate (Co (NO_3_)_2_.6H_2_O), and diphenylamine, in solution of water and ethanol (1:1v/v ratio). The Co-DPA was characterized by XRD, and FTIR. A wastewater sample was collected from Gurage zone Butajira town around textile fabric from Akamuj river, Ethiopia. Heavy metals removal capacity of Co-DPA was determined, and batch experiments were conducted to look at the effects of pH, dose, initial metal concentration, and contact time on the adsorptions of heavy metals. The effect of condition parameters like concentration of lead, chromium, cadmium, and contact time was studied to obtain the optimum condition with high removal efficiency. The measurement of the concentration of heavy metals before and after treatment with the prepared Co-DPA was done by AAS.

## Materials and methods

### Materials

All chemicals and reagents procured were of analytical grade and used without additional purification. Cobalt (II) nitrate hexahydrate, ethanol, diphenylamine, hydrochloric acid, sodium hydroxide, Nitric acid, Perchloric acid, Lead (II) chloride, Cadmium (II) chloride, and Chromium (III) chloride were purchased from Sigma Aldrich. Following a minimum 24-h soak in 10% Nitric acid, all vessels were cleaned by being repeatedly washed with deionized (DI) water. Real samples of heavy metal ions (Cd^2+^, Pb^2+^, and Cr^3+^) with metal concentrations as 0.267, 0.075, and 0.125 mg/L respectively were collected from Gurage zone Butajira town around textile fabric from Akamuj river, Ethiopia. The standard solution of Cd, Pb, and Cr were prepared by dissolving metal precursors in distilled water.

### Synthesis of cobalt-complex

Cobalt complex (Co-DPA) was synthesized via facile approach using cobalt (II) nitrate hexahydrate (Co (NO_3_)_2_.6 H_2_O). 24.69 g of cobalt nitrate hexahydrate was dissolved in 5 mL of distilled water, and 5 g of diphenylamine was dissolved in 5 mL of ethanol. The two solutions were combined and swirled on a hot plate magnetic stirrer for an hour to create a homogenous solution. The mixture was then preserved for 3 days, and a reddish-pink precipitate was formed. The formed precipitate was filtered by filter paper using suction filtration and dried in a drying oven at 40 °C for 24 h. The attained precipitate, named Co-DPA subsequently, was retained for further characterization and experiments.

### Characterization

Powder X-ray diffraction (PXRD) technique was used to study the purity and crystallinity of the bulk material (Shimadzu XRD- 7000). FT-IR (Fourier-transform infrared) spectroscopy was used to examine the surface functional groups of the Co-DPA complex (PerkinElmer spectrometer). A DW-AA320N Atomic Absorption spectrometer (AAS) was used for metal adsorption studies.

### Collection of waste-water sample

Wastewater was collected from Gurage zone, Butajira town, Ethiopia from the car wash labajo area and transported to Chemistry laboratory, Hawassa University, Ethiopia. The sample was fixed by adding 3 drops of 69% nitric acid and stored at 4 ℃ for heavy metals determination before and after the treatment. The actual concentrations of heavy metals were measured by using AAS, before being treated by Co-DPA.

### Batch adsorption experiment to optimize adsorption parameters

Batch adsorption tests were performed to observe the effect of pH, initial metal ions concentration, adsorbent dosage, adsorption kinetics, adsorption isotherms, and period of mixing needed to attain equilibrium for Pb(II), Cr(III), and Cd(II) adsorbed by Co-DPA.

### pH study

To determine the effect of pH on the removal of heavy metals from wastewater, the metals sample working solution was prepared. 20 mg/L metals concentration and 3 g/L of Co-DPA were added to the pH optimized as 3, 5, 7, 9 and 11, then centrifuged for 30 min and the supernatant was used to measure heavy metals concentration.

## Results and discussion

### Characterization of Co-DPA

**Fig. 1:**
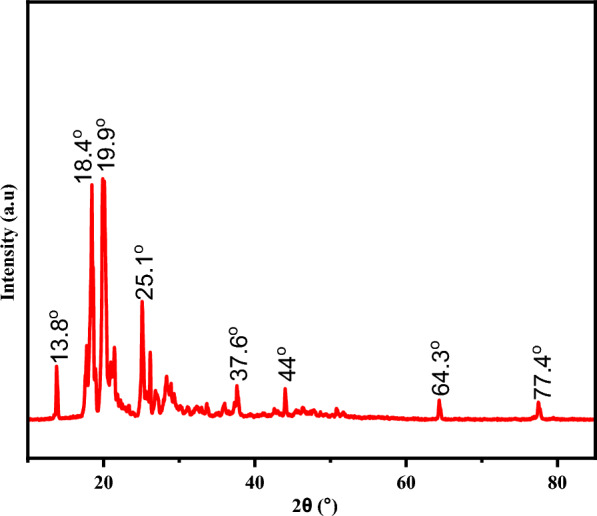
PXRD graph of synthesized Co-DPA

Powder X-ray diffraction (XRD) analysis was considered to examine the structure and crystallinity of the samples (Fig. 1). The intensity of the detected rays about scattering angle two theta (2θ) was used to ascertain the crystalline nature of the as-synthesized Co-DPA. The crystallite domain size (grain size) was calculated from the width of the peaks using Scherrer’s formula. As shown in Fig. 1, the calculated grain size, from average of the tallest peak, at an angle of 2θ is 34.55 nm. The calculated grain size (mean diameter of the particle) is greater than 2 nm and less than 50 nm, which is the characteristic nature of mesoporous materials from Scherrer’s equation [67].

**Fig. 2:**
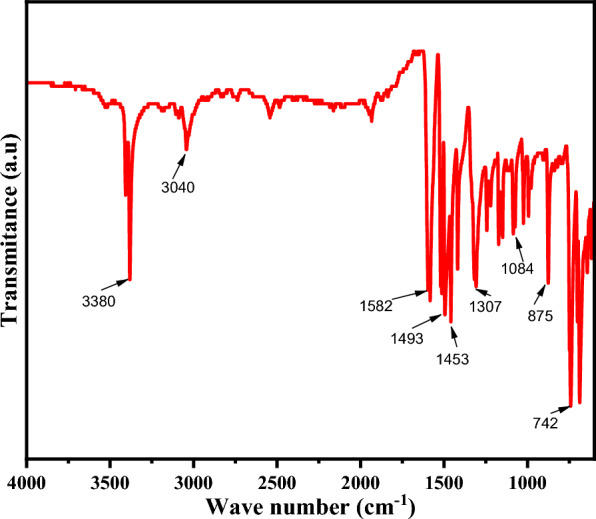
FT-IR graph of synthesized Co-DPA

The FTIR spectra were acquired using a Perkin Elmer FT-IR spectrometer in transmission mode. With a resolution of 4 cm^−1^ (Fig. 2), each sample in the KBr mix was scanned 64 times from 4,000 to 400 cm^−1^. The secondary N–H vibration at frequencies (3380 and 3040 cm^−1^) after complex formation showing coordination of the amine nitrogen atom. The peaks appeared around 1582, 1493, and 1453 cm^−1^ is assigned for the C=C stretching vibrations for the aromatic rings of the diphenylene ligand coordinated to the metal center. The strong searching vibration peak appeared around 742 and 688 cm^−1^ are confirmed the coordination of Co-with N of the diphenylene ligand.

### Optimization of adsorption parameters

Adsorption behaviour was analyzed by a batch method, where factors that affect the process of adsorption such as pH, adsorbent dose, initial metals concentration, and contact time were investigated. The standard solution of Pb, Cd, and Cr were prepared by dissolving metal precursors in distilled water.

#### Effect of pH

**Fig. 3:**
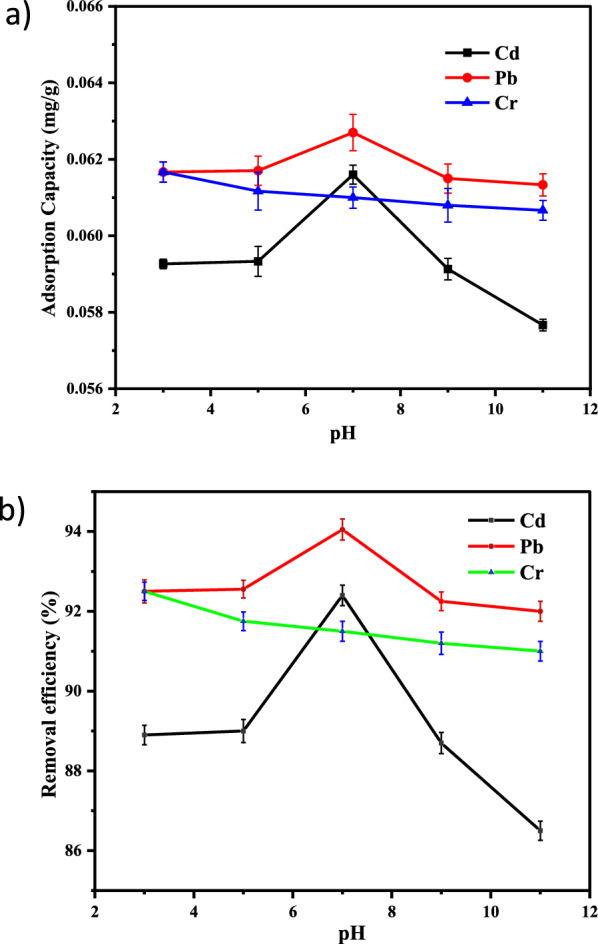
Graphical representation of the impact of initial pH on **a** adsorption capacity and **b** removal efficiency (with error bar) of Cd^2+^, Pb^2+^, and Cr^3+^ by Co-DPA (error bars represent ± standard experimental errors)

For adsorbent Co-DPA the effect of pH on the removal efficiency of heavy metals was considered from a range of 3 to 11 under the precise conditions (at a contact time of 30 min, 20 mg/L adsorbate solution with 3 g of the adsorbent and temperature of 25 °C with an agitation speed of 150 rpm). The percentage of Pb(II) and Cd(II) adsorbed by Co-DPA first increases with an increase in pH from pH 3–7 and then decreases later (Fig. 3a). For Cr(III) ion, the maximum removal efficiency obtained is at pH 3. The maximum percent removal of heavy metal ions was obtained at pH 7 for Cd and Pb and at pH 3 for Cr, which is 92.4%, 94.05%, and 92.5%, respectively (Fig. 3b). For Lead high removal efficiency obtained at pH 7 is the same as the results obtained by Li et al. on the removal of chromium from wastewater by Mg-Loaded Biochars with the removal efficiency of 87% partaking the maximum adsorption capacity of 2.7 mg/g [68]. The high removal efficiency of cadmium obtained at pH 7 is the same as the result obtained by Bayuo et al. [69] on the removal of heavy metals from wastewater using shea fruit shell biomass. Most of the adsorption space for the elimination of cadmium and lead could be occupied by protons, at lower pH levels (pH 3.0). The proton attached is released from active sites at moderate pH, and the quantity of adsorbed metal rises. The metal precipitate can occur at high pH levels (pH > 7), which reduces adsorption. Cation exchange and surface complexation are two potential explanations for why metal’s adsorption capacities increase when pH rises. The delayed adsorption at high pH is results from slow diffusion into the adsorbent's bulk and an electrostatic barrier between positively charged adsorbate species that have been adsorbed onto the surface of the adsorbent and cationic adsorbate species that are present in the solution [70]. Moreover, the unique nature of chromium and the adsorbent surface explains the high adsorption capacity of metallic chromium on an adsorbent surface at lower pH. At high pH value Cr(III) adsorption onto Co-DPA decreases as metal ions react with OH¯ ions and gets precipitated as a metal hydroxide. As a result, this pH was chosen as the ideal value for the remaining studies [71–73].

#### Effect of adsorbent dosage

**Fig. 4:**
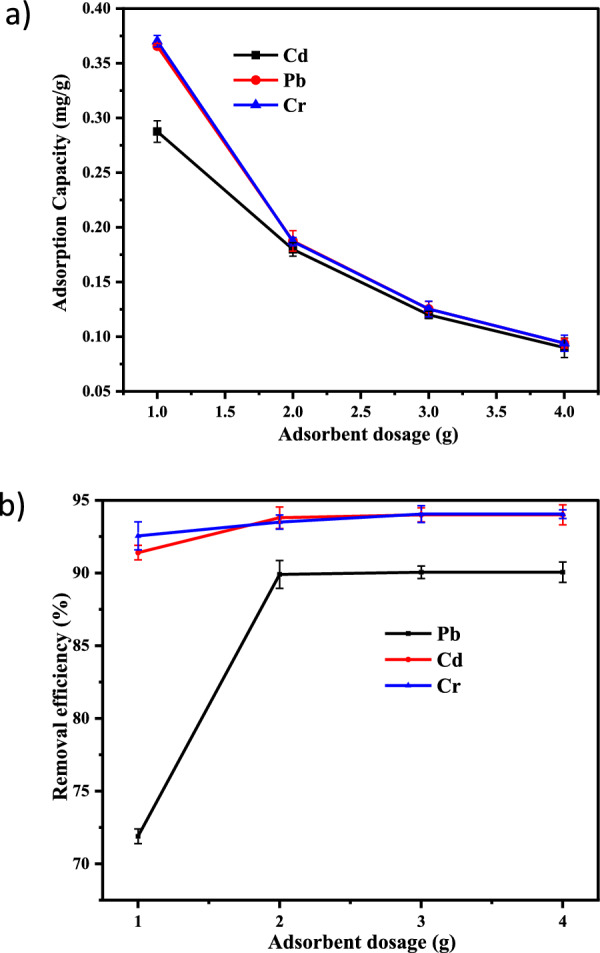
Graphical illustration of the impact of adsorbent dosage on **a** adsorption capacity  **b** removal efficiency (with error bar) of Cd^2+^, Pb^2+^ and Cr^3+^ by Co-DPA (error bars represent ± standard experimental errors)

The impact of the adsorbent dose was analyzed by the variable quantity of Co-DPA from 1 to 4 g and kept other parameters constant (pH 3 for Cr and pH 7 for Cd and Pb, 20 mg/L adsorbate concentration, 30 min contact time). As shown in Fig. 4a, the adsorption capacity was found to be highest at the lowest adsorbent concentration and reduced as the dose rose. In this work, As the adsorbent dose increased, the adsorption capacity declined, which was attributed to adsorption sites staying unsaturated throughout the adsorption reaction due to the increased number of accessible adsorption sites favoring enhanced metal ion uptake. Adsorption dosage increased due to the availability of a greater surface area and adsorption sites for a constant amount of metal ions, and accessible sites would overlap [73, 74].

When the adsorbent dose was increased from 1 to 4 g, the percentage of Cr (III), Cd (II), Pb(II) removal was increased (Fig. 4). As the dosage amount increased from 1 to 3, the removal efficiency rose from 71.9% to 90.05% for Pb^2+^, from 91.4 to 94% for Cd^2+^, and 92.55 to 94 0.05% for Cr^3+^. The superior removal efficiency was obtained when the adsorbent dosage was 3 g i.e., 90.05%, 94%, and 94.05%, respectively. No change was observed when dosage amount increased to 4 g (Fig. 4b). The higher number of accessible adsorption sites encouraged the enhanced uptake of the metal ions which is the cause of the constant adsorption capacity with increasing doses of adsorbent [48, 74].

#### Effect of initial metals concentration

**Fig. 5:**
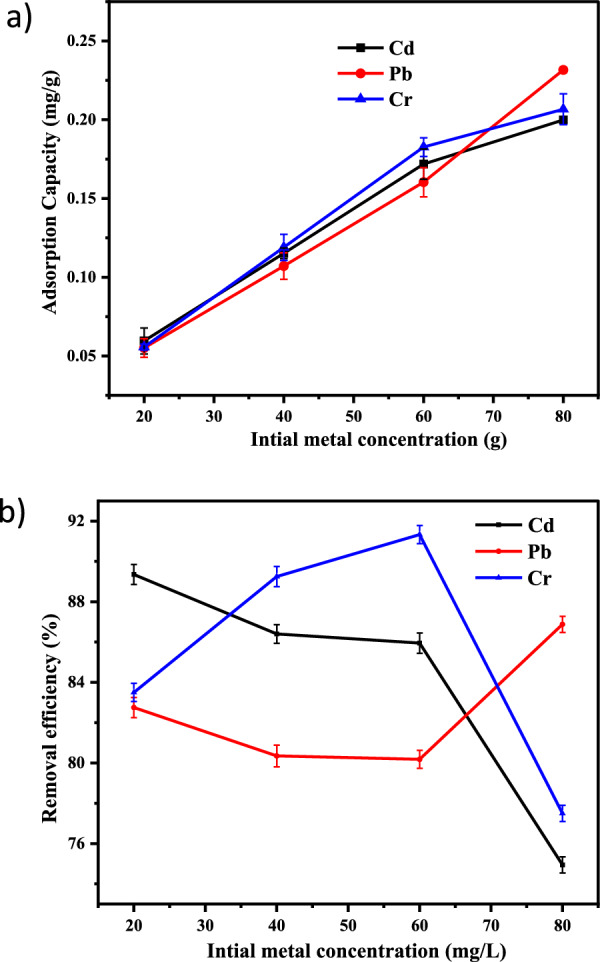
Representation of the impact of initial metals concentration on **a** adsorption capacity and **b** removal efficiency of Cd^2+^, Pb^2+^ and Cr^3+^ by Co-DPA with error bars (error bars represent ± standard experimental errors)

Metal ions standard solutions in the following concentrations: 20, 40, 60, and 80 mg/L were generated, and the other parameters kept similar as before. Co-DPA was used to treat the solutions, and an AAS was used for analysis. The % removal varied consistently as the starting concentration of heavy metal ions changed. For Cr (III), Pb(II), and Cd(II), the percent removal for metal ions falls as the initial concentration slowly rises to 60 mg/L, then declines rapidly as the initial concentration rises to 80 mg/L. The adsorption capacity increased as the concentration of metals increased (Fig. 5a). It is evident from the Figure that the percentage of Pb^2+^, Cd^2+^, and Cr^3+^ removal efficiency decreased from 89.35% to 74.9475% for Cd^2+^, 82.75% to 77.5% for Pb^2+^, 90.55% to 84.375% for Cr^3+^ with the increase in initial concentration of metal ions (Fig. 5b). Different heavy metal ions may have different chemical affinities and ion exchange capacities concerning the chemical functional group on the surface of the adsorbent, which can explain differences in the percentage of different heavy metal ions eliminated at the same initial metal ions concentration, dose, and contact duration [30, 47, 48, 74, 75].

#### Effect of contact time

**Fig. 6:**
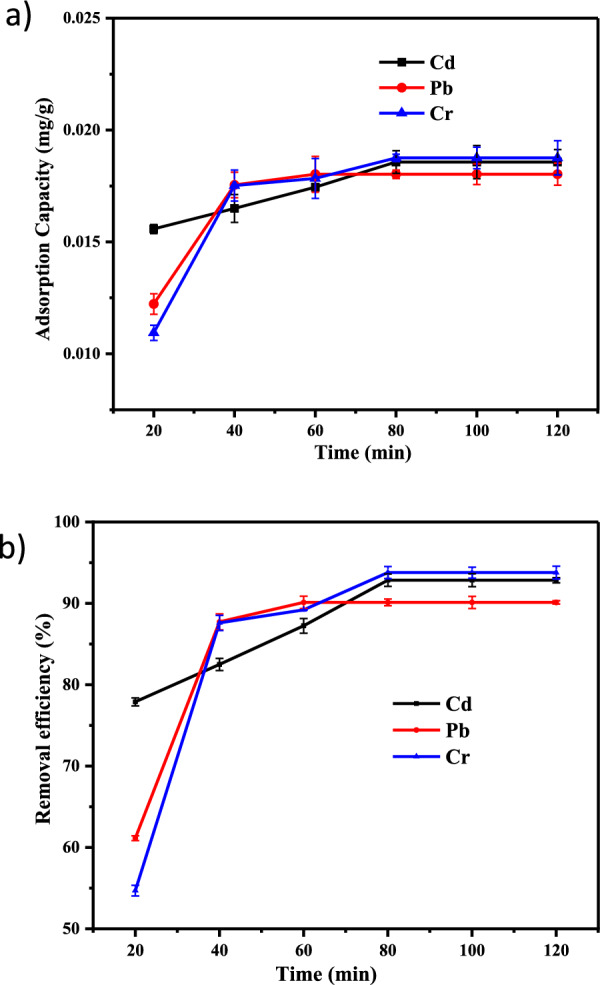
Graphical representation of the impact of contact time on **a** adsorption capacity and **b** removal efficiency of Cd^2+^, Pb^2+^ and Cr^3+^ by Co-DPA with error bars (error bars represent ± standard experimental errors)

The effects of contact time Pb(II), Cd(II), andCr (III) adsorption on Co-DPA at different adsorption times (20, 40, 60, 80, 100, and 120 min) is shown in (Fig. 6), and pH 3 for Cr (III) and 7 for Cd(II) and Pb(II), adsorbent dose 3 g, initial concentration was 60 mg/L. The initial adsorption was highly rapid, and as the adsorption duration increased, the sorption kinetics increased gradually until the adsorption eventually hit equilibrium in 80 min in the case of Cd^2+^ and Cr^3+^ with a removal efficiency of 92.85 and 93.785%, respectively (Fig. 6). Pb^2+^ had a high removal efficiency of 90.13% at 60 min, matching the findings of Kowsura et al. [76]. Cadmium (II) adsorption by Co-DPA has maximum removal efficiency of 92.85% which is the same as the result obtained by [74] with a maximum adsorption capacity of 33 mg/g at 80 min. During the adsorption process at the initial stage, many vacant sites are available for adsorption. As adsorption time progresses the active sites are occupied by adsorbates which make constant adsorption efficiency. This may be clarified by the fact that originally, the ion absorption rate was higher since entire sites on the adsorbent were unoccupied and the concentration of ions was high, but that rate remained constant as all sites were filled consequently because doing so prevents the additional adsorbate from being taken up [30, 34, 48].

#### Adsorption experiment for real sample

The collected wastewater sample was brought to the laboratory for the adsorption experiment and digested with 4 mL nitric acid and 2 mL perchloric acid for 80 min at a temperature of 120 °C. 20 mL of digested sample was taken, and 3 g of the synthesized Co-DPA was added and allowed to shake on a reciprocating shaker for 80 min to determine Pb, and 3 g of the synthesized Co-DPA was added and allowed to shake on a reciprocating shaker for 80 min to determine Cd^2+^ and Cr^3+^. The sample was filtered by filter paper and brought to measure heavy metals concentration by AAS. The removal efficiency and adsorption capacity were estimated. The metal ions concentration before being treated with synthesized Co-DPA recorded for Cd^2+^, Pb^2+^, and Cr^3+^ was 0.267 mg/L, 0.075 mg/L, and 0.125 mg/L, respectively. After being treated with the adsorbent the concentration of the metal was obtained as 0.0129, 0.00028, and 0.00054 for Cd^2+^, Pb^2+^, and Cr^3+^, respectively. For Cd^2+^, Pb^2+^, and Cr^3+^, the removal efficiencies of the adsorbent for heavy metals in actual samples were determined to be 95.6%, 99.5%, and 99.5% respectively.

#### Recovery studies

**Fig. 7:**
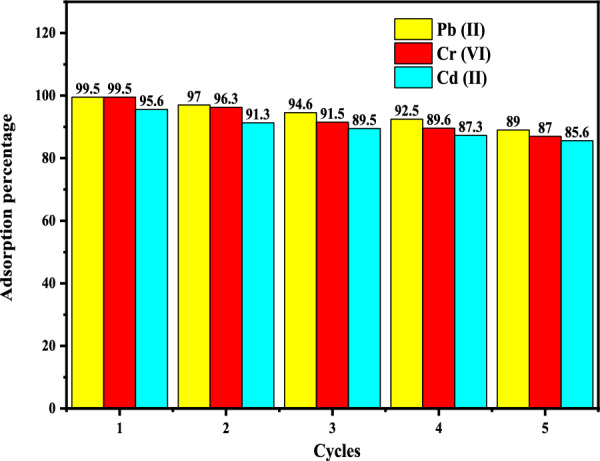
Illustration of the percent removal of heavy metal ions from real wastewater sample along with the reusability studies for Co-DPA complex

Reusing the adsorbent by regenerating its adsorption characteristics is an economic necessity in many applications. With growing raw material and wastewater treatment process expenses, the allure of product recovery technologies has grown dramatically [74, 77]. The Cu-DPA adsorbent was evaluated for its reusability. The recycling results shown in Fig. 7 show that it maintains its activity despite a decline in metal ion removal efficiency, and it is pH dependent and performed by adding HCl and NaOH to solution.

In a typical experiment, 0.01 M HNO_3_ or HCl and 0.005 M NaOH eluents were added to the solution to perform the recycling test. Metals were initially adsorbed on Cu-DPA from 60 mL solutions containing 80 mg/L metal ions at pH 3 (Cr) and 7 (Cd &Pb). The Cu-DPA were then stripped with 30 mL eluent while agitating at 25 °C for 30 min. The Cu-DPA complex was separated, and the metal ion concentration in the supernatant was determined. The adsorption–desorption cycles were done three times for each measurement.

#### Adsorption isotherms

**Fig. 8:**
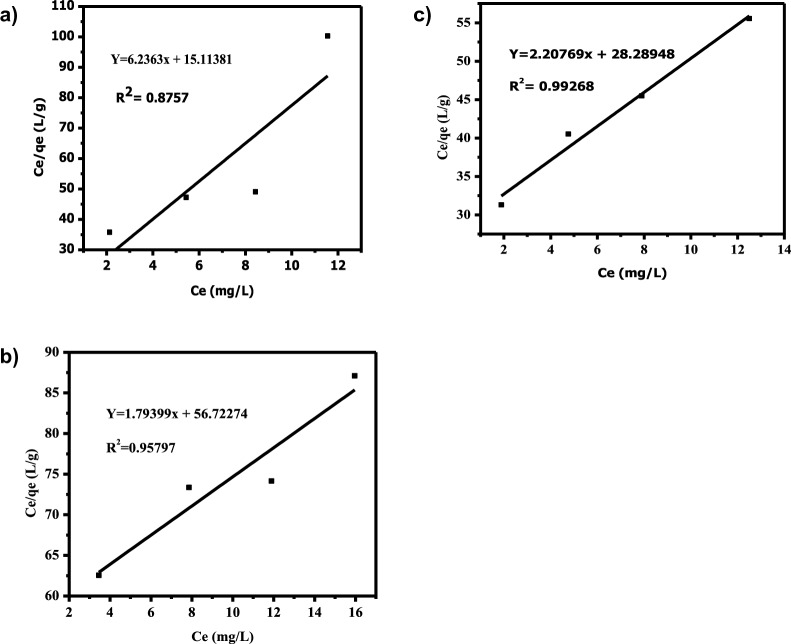
Langmuir adsorption isotherm for **a** Cd^2+^, **b** Pb^2+^, and **c** Cr^3+^

Figure 8 demonstrates the adsorption isotherm with fitting models. Adsorption isotherms are often used to explain equilibrium studies that provide the adsorbent's capacity and the equilibrium interactions between adsorbent and adsorbate [30, 36, 44, 78–80]. Adsorption isotherms are the fraction of the quantity adsorbed and remaining in solution at set temperature as equilibrium. The Langmuir and Freundlich isotherms are the original and most basic proven relations that illustrate the adsorption equation. The fundamental tenet of the Langmuir hypothesis is that once a metal molecule occupies a particular homogenous location inside the adsorbent, no further adsorption can take place there. A description of the Langmuir isotherm model is stated in Eq. (1).

1 $$\frac{{\varvec{C}}{\varvec{e}}}{{\varvec{q}}{\varvec{e}}}=\frac{1}{{{\varvec{K}}}_{{\varvec{L}}}{\varvec{q}}{\varvec{m}}}+\boldsymbol{ }\frac{{\varvec{C}}{\varvec{e}}}{{\varvec{q}}{\varvec{m}}}.$$

where q_m_ symbolizes the maximum monolayer adsorption capacity (mg/g), KL stands for Langmuir constant associated with the energy of adsorption (L/g), C_e_ and q_e_ are the equilibrium concentration (mg/L), and adsorption capacity (mg/g) respectively. The equation above produces a straight line with a slope of 1/q_m_ and an intercept of 1/K_Lqm_ when plotting C_e_/q_e_ vs C_e_ (Fig. 8).

**Fig. 9:**
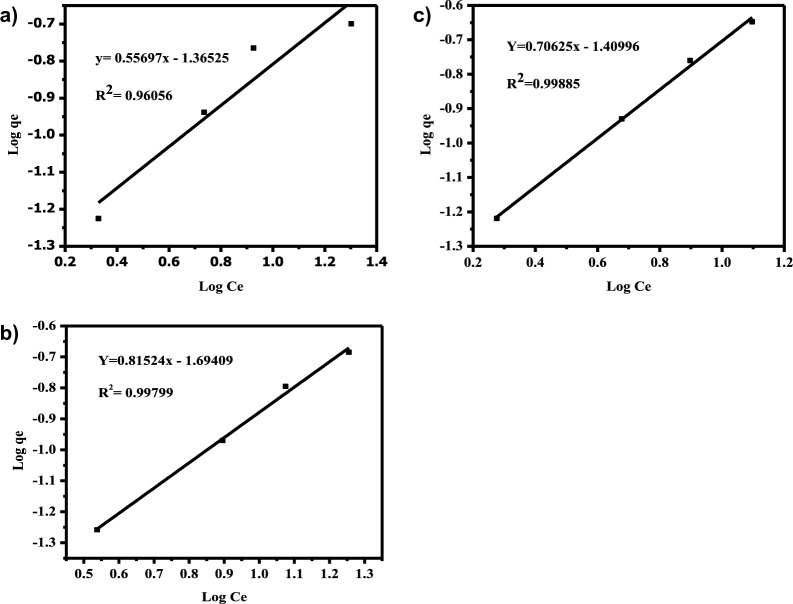
Freundlich adsorption isotherm for **a** Cd^2+^, **b** Pb^2+^ and **c** Cr^3+^

There are interactions between molecules that have been adsorbed and heterogeneous surface energy systems in multilayer adsorption, which is expressed by the Freundlich isotherm model. Equation (2) used to describe the Freundlich isotherm. In this case, the Freundlich isotherm model's constants for sorption capacity (K_F_) and sorption intensity (1/n) are involved, and the exponent (1/n) indicates the system's favorability and capacity. Comparing log qe to log Ce from the previous equation yields a straight line with a slope of 1/n and an intercept of log K_F_ (Fig. 9). The slope 1/n, which has a value between 0 and 1, is a measure of surface heterogeneity or adsorption intensity, which turn additionally heterogeneous because its value attempts to zero. Normal Langmuir isotherm is designated by a value of 1/n below one, and cooperative adsorption is implied by a value of 1/n above one [81]. The K_F_ is an assessment of adsorption capacity increased with concentration for Pb^2+^, Cd^2+^, and Cr^3+^ adsorption. The magnitude of the exponent n shows the favorability and capacity of the adsorbent/adsorbate system. 1/n for Cd^2+^, Pb^2+^, and Cr^3+^ is 0.79005, 0.880, and 0.74007, respectively. These values are the measure of favorability of the adsorption of adsorbate on the heterogeneous surface of the adsorbent [32, 69, 71]. Karthikeyan et al. have stated that the favorable adsorption condition is represented through n values between 1 and 10.

2 $${\varvec{log}}\,{\varvec{qe}}={\varvec{log}}{{\varvec{K}}}_{{\varvec{F}}}+\frac{1}{{\varvec{n}}}{\varvec{log}}{\varvec{C}}{\varvec{e}}$$

To determine the favorable or unfavorable behavior of an adsorption system, Hall et al. [82] introduced a dimensionless separation factor or equilibrium parameter, RL, as a crucial component of the Langmuir isotherm, which is specified as in Eq. (3).

3 $$RL = 1/1+KLCo$$

where *co* represents the adsorbate’s initial concentration (mg/L), KL defines Langmuir constant (L/mg). If *RL* > 1.0, the sorption isotherm remains unfavorable; = 1.0 (linear); 0 < *RL* < 1.0 (favorable) and *RL* = 0 (irreversible) [83]. The achieved findings (*RL* = 0.357, 0.436, 0.271 for Cd^2+^, Pb^2+^ and Cr^3+^, respectively) confirms that the sorption of Cd^2+^, Pb^2+^ and Cr^3+^ onto the Co-DPA was favorable.

Table 2 lists the isotherm parameters for the Pb(II), Cd(II), and Cr(III) adsorption in the Langmuir and Freundlich models. The values of R2 of the Langmuir model were lower than Freundlich isotherm model R2 (Cd^2+^(0.995), Pb^2+^(0.997), Cr^3+^(0.996))*,* from which it was concluded that the adsorption of Pb(II), Cd(II) and Cr(III) ions fitted better in the Freundlich isotherm model. This showed that the adsorption of Cr(III), Pb(II), and Cd(II) was directed by multilayer adsorption on a heterogeneous surface of an adsorbent. The n value greater than 1 (n > 1) is documented as an L-type isotherm indicating significant affinity among adsorbate and adsorbent and revealing chemisorption. The Freundlich adsorption capacity (KF) reveals the removal of heavy metal ions from wastewater facilely. The greater the adsorption intensity the higher the K_F_ value.

**Table 2:** Analyzed isotherm parameters for the adsorption of Cd^2+^, Pd^2+^ and Cr^3+^ on to Co-DPA

Isotherm model	Adsorbate		
	Cd	Pb	Cr
Langmuir			
q_m_ (mg/g)	0.1604	0..557	0.453
k_L_(L/mg)	0.4125	0.032	0.078
R_L_	0.1081	0.6098	0.379
R^2^	0.8757	0.95797	0.99269
Freundlich			
N	1.795	1.227	1.416
K_F_(L/g)	0.04313	0.02023	0.039
R^2^	0.96056	0.99799	0.99885

The fraction of the Co-DPA surface covered by Cd^2+^, Pd^2+^, and Cr^3+^ was calculated from the optimized concentration using Eq. (4).

4 $$\uptheta =1 -\frac{Ce}{ Co}$$

where θ is a fraction of surface coverage, Co is the initial metal ions concentration and Ce is the equilibrium metal ions concentration. The calculated fraction of surface coverage during the adsorption of Cd^2+^, Pd^2+^, and Cr^3+^ onto Co-DPA is presented in Table 3. The fraction of Co-DPA surface covered by Cd^2+^, Pd^2+^, and Cr^3+^ was obtained as 0.8935, 0.8275, and 0.835 for Cd^2+^, Pd^2+^, and Cr^3+^ respectively. These values indicate that 89.35%, 82.75%, and 83.5% of the pore space of Co-DPA is covered by metal ions which is the same as the result obtained on the removal of lead from wastewater by Co-DPA.

**Table 3:** The fraction of the Co-DPA surface covered by Cd^2+^, Pd^2+^, and Cr^3+^

Metal ions	Metal ions surface coverage
Cd^2+^	0.8935
Pd^2+^	0.8275
Cr^3+^	0.8350

The number of hopping (n) an adsorbate makes, as determined by Eq. (5), may be associated with the probability that it will discover an empty site on the adsorbent surface during sorption (5).

5 $${\text{n}}=\frac{1}{(1-\theta )\theta }.$$

where n is the number of hopping done by the adsorbate and θ is the surface coverage. By using Eq. (5) the number of hopping done by adsorbate to find vacant sites on Co-DPA was obtained as 10.504, 6.993, and 7.246 for Cd^2+^, Pd^2+^, and Cr^3+^, respectively.

#### Adsorption kinetics

**Fig. 10:**
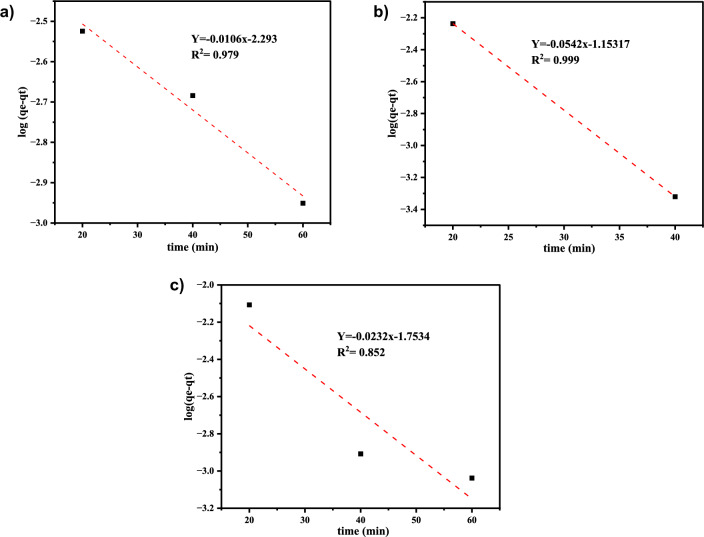
Pseudo-first orders kinetic model of **a** Cd^2+^, **b** Pb^2+^, and (c) Cr^3+^

The kinetics of cadmium, lead, and chromium adsorption onto Co-DPA were investigated using the pseudo-second order and pseudo-first order kinetic models to suggest which model the sorption system follows. This can be described by models of pseudo-first order and pseudo-second order. Scientifically pseudo first order is stated as in Eq. (6):

6 $${\varvec{log}}\,\left({\varvec{qe}}-{\varvec{qt}}\right)={\varvec{log}}\,{\varvec{qe}}-\frac{{\varvec{K}}1}{2.303}\mathbf{t}$$

where qt expresses the adsorption capacity at time t, qe symbolizes the equilibrium adsorption capacity (mg/g), and K1 means the pseudo-first-order rate constant (g/mg) which can be estimated by plotting between log (qe-qt) and t (Fig. 10).

**Fig. 11:**
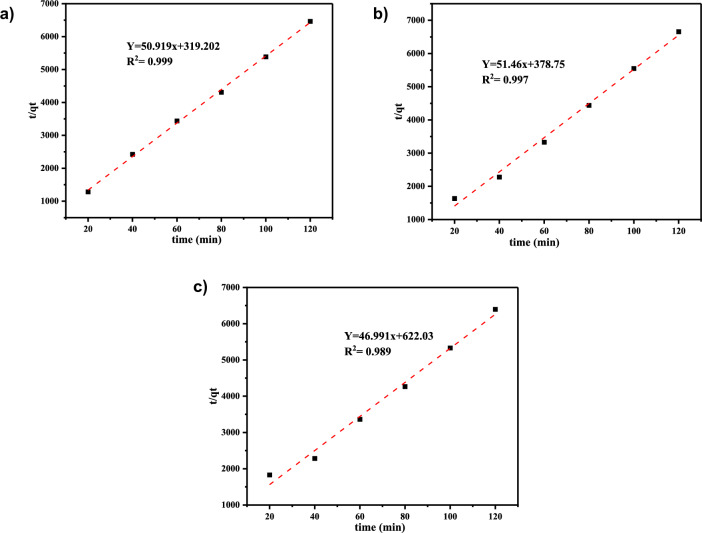
Pseudo-second order kinetic model of **a** Cd^2+^, **b** Pb^2+^, and **c** Cr^3+^

Pseudo-second order kinetic model is stated mathematically as in Eq. (7):

7 $$\frac{t}{{q_{t} }} = \frac{1}{{K_{2} qe^{2} }} + \frac{1}{{q_{e} }}{\text{t}}$$

where K_2_ expresses the rate constant for pseudo-second order (g mg^−1^ min^−1^). The value of q_e_ was estimated through the plot between t/qt and t (Fig. 11).

From the pseudo-first and second order kinetic model linear plotted with different adsorption time and kinetic model constants like k1, k2, and qe values were determined from the slope and intercept of plots, are represented in Table 3. Table 3 reveals that the linear plot of Cd^2+^ and Cr^3+^ shown in Figs. 10 and 11 has a higher R2 value in the pseudo-second order. This shows that the kinetics data suit perfectly with the pseudo-second-order model. The computed values of qe almost matched with the experimental data obtained from the pseudo-second-order kinetics in addition to the larger values of R2 (Table 4). Also, it was noted that the values of k1 were higher than the comparable values of k2. The best fit for the Pb^2+^ adsorption studies were therefore believed to be the pseudo first-order kinetic model. The kinetics results from various investigations were also said to fit the pseudo-second-order kinetic model quite well; including the adsorption of Cd^2+^ ions on Jatropha peel, adsorption of Pb^2+^ ions on pumpkin seed shell activated carbon, adsorption of Pb^2+^ ions on Co-DPA, and adsorption of Cr^3+^ ions on cooked tea dust [43].

**Table 4:** Calculated kinetic parameters for the adsorption of Cd^2+^, Pb^2+^,  and Cr^3+^ on to Co-DPA

Kinetic model								
Pseudo first order					Pseudo second order			
Adsorbate	K1 (min^−1^)	q_exp_ (mg/g)	qe_cal_ (mg/g)	R^2^	K_2_ (g mg^−1^mi^−1^)	q_exp_ (mg/g)	qe_cal_ (mg/g)	R^2^
Cd^2+^	0.025	0.01857	0.0051	0.9897	8.16	0.01857	0.02	0.994
Pb^2+^	0.125	0.018026	0.0294	-0.998	6.99	0.018026	0.02	0.997
Cr^3+^	0.054	0.018757	0.0176	0.923	3.54	0.018757	0.021	0.995

## Conclusion

The Co-DPA complex was easily produced from cobalt (II) nitrate hexahydrate and diphenylamine linker. Co-DPA was evaluated for its efficacy in removing heavy metal ions from wastewater and its adsorption capacity. From this study, it is concluded that the Co-DPA is an excellent adsorbent for the removal of the heavy metal ions such as Cd^2+^, Pb^2+^, and Cr^3+^ from wastewater samples. The effect of adsorption factors such as contact time, pH value, initial metals concentration, and adsorbent (synthesized Co-DPA) dose on adsorption capacity and removal efficiency was examined. The removal efficiency was recorded as 95.6%, 99.5%, and 99.5% for the Cd^2+^, Pb^2+^, and Cr^3+^, respectively. More importantly, the adsorption isotherm was governed by Freundlich isotherm and adsorption mechanism was pseudo-first order for Pb^2+^ and pseudo-second order for Cd^2+^, and Cr^3+^. This suggests that a multilayer has formed on the surface of the adsorbent. Therefore, the synthesized Co-DPA complex could be a potential candidate for the massively efficient adsorptive removal of heavy metal ions from wastewater.

## Data Availability

The datasets used and/or analyzed in this study are accessible in the publication and can be obtained upon request from the corresponding author.

## References

[1] Gorchev HG, Ozolins G (1984). WHO guidelines for drinking-water quality. WHO Chron.

[2] Ahluwalia SS, Goyal D (2007). Microbial and plant derived biomass for removal of heavy metals from wastewater. Biores Technol.

[3] Ke F, Qiu L-G, Yuan Y-P, Peng F-M, Jiang X, Xie A-J (2011). Thiol-functionalization of metal-organic framework by a facile coordination-based postsynthetic strategy and enhanced removal of Hg2+ from water. J Hazard Mater.

[4] Dhakshinamoorthy A, Alvaro M, Garcia H (2011). Aerobic oxidation of styrenes catalyzed by an iron metal organic framework. ACS Catal.

[5] Kothapalli CR (2021). Differential impact of heavy metals on neurotoxicity during development and in aging central nervous system. Curr Opin Toxicol.

[6] Mitra S, Chakraborty AJ, Tareq AM, Emran TB, Nainu F, Khusro A (2022). Impact of heavy metals on the environment and human health: novel therapeutic insights to counter the toxicity. J King Saud Univ Sci.

[7] Mohanty S, Benya A, Hota S, Kumar MS, Singh S (2023). Eco-toxicity of hexavalent chromium and its adverse impact on environment and human health in Sukinda Valley of India: a review on pollution and prevention strategies. Environ Chem Ecotoxicol.

[8] Zhong G, Lu S, Chen R, Chen N, Tan Q-G (2020). Predicting risks of cadmium toxicity in salinity-fluctuating estuarine waters using the Toxicokinetic-Toxicodynamic Model. Environ Sci Technol.

[9] Hui C, Guo Y, Li H, Gao C, Yi J (2022). Detection of environmental pollutant cadmium in water using a visual bacterial biosensor. Sci Rep.

[10] Kubier A, Wilkin RT, Pichler T (2019). Cadmium in soils and groundwater: a review. Appl Geochem.

[11] Turner MC, Andersen ZJ, Baccarelli A, Diver WR, Gapstur SM, Pope CA (2020). Outdoor air pollution and cancer: an overview of the current evidence and public health recommendations. CA A Cancer J Clin.

[12] Rousseau M-C, Parent M-E, Nadon L, Latreille B, Siemiatycki J (2007). Occupational exposure to lead compounds and risk of cancer among men: a population-based case-control study. Am J Epidemiol.

[13] Raouf Ms A, Raheim AR (2016). Removal of heavy metals from industrial waste water by biomass-based materials: a review. J Pollut Eff Cont..

[14] Yari M, Norouzi M, Mahvi AH, Rajabi M, Yari A, Moradi O (2016). Removal of Pb(II) ion from aqueous solution by graphene oxide and functionalized graphene oxide-thiol: effect of cysteamine concentration on the bonding constant. Desalin Water Treat.

[15] Bernal V, Giraldo L, Moreno-Piraján JC (2021). Physicochemical parameters of the methylparaben adsorption from aqueous solution onto activated carbon and their relationship with the surface chemistry. ACS Omega.

[16] Geng H, Xu Y, Zheng L, Gong H, Dai L, Dai X (2020). An overview of removing heavy metals from sewage sludge: achievements and perspectives. Environ Pollut.

[17] Gupta S, Benien P, Sahoo PK (2010). Ion exchange resins transforming drug delivery systems. Curr Drug Deliv.

[18] Prifti H, Parasuraman A, Winardi S, Lim TM, Skyllas-Kazacos M (2012). Membranes for redox flow battery applications. Membranes.

[19] Lee H (2002). Characterization of anion exchange membranes fouled with humate during electrodialysis. J Membr Sci.

[20] Garba MD, Usman M, Mazumder MAJ, Al-Ahmed A, Inamuddin (2019). Complexing agents for metal removal using ultrafiltration membranes: a review. Environ Chem Lett.

[21] Al-Ghouti MA, Da’ana D, Abu-Dieyeh M, Khraisheh M (2019). Adsorptive removal of mercury from water by adsorbents derived from date pits. Sci Rep.

[22] Mohammad A, Ansari SN, Chaudhary A, Ahmad K, Rajak R, Tauqeer M (2018). Enthralling adsorption of different dye and metal contaminants from aqueous systems by cobalt/cobalt oxide nanocomposites derived from single-source molecular precursors. ChemistrySelect.

[23] Sarkar S, Adhikari S, Jana BB, Mandal RN, Jayasankar P (2018). Adsorption technique for removal of heavy metals from water and possible application in wastewater-fed aquaculture. Wastewater management through aquaculture.

[24] Syahirah Kamarudin N, Jusoh R, Dina Setiabudi H, Fateha Sukor N, Haslinda SJ (2021). Potential nanomaterials application in wastewater treatment: physical, chemical, and biological approaches. Mater Today Proc.

[25] Mahmoudi MM, Nasseri S, Mahvi AH, Dargahi A, Khubestani MS, Salari M (2019). Fluoride removal from aqueous solution by acid-treated clinoptilolite: isotherm and kinetic study. DWT.

[26] Priyadarshanee M, Das S (2021). Biosorption and removal of toxic heavy metals by metal tolerating bacteria for bioremediation of metal contamination: a comprehensive review. J Environ Chem Eng.

[27] Jiang J-Q, Ashekuzzaman S (2012). Development of novel inorganic adsorbent for water treatment. Curr Opin Chem Eng.

[28] Asiabi H, Yamini Y, Shamsayei M, Tahmasebi E (2017). Highly selective and efficient removal, and extraction of heavy metals by layered double hydroxides intercalated with the diphenylamine-4-sulfonate: a comparative study. Chem Eng J.

[29] Jha MK, Joshi S, Sharma RK, Kim AA, Pant B, Park M (2021). Surface modified activated carbons: sustainable bio-based materials for environmental remediation. Nanomaterials.

[30] Pourali P, Rashtbari Y, Behzad A, Ahmadfazeli A, Poureshgh Y, Dargahi A (2023). Loading of zinc oxide nanoparticles from green synthesis on the low cost and eco-friendly activated carbon and its application for diazinon removal: isotherm, kinetics, and retrieval study. Appl Water Sci.

[31] Almasi A, Omidi M, Khodadadian M, Khamutian R, Gholivand MB (2012). Lead(II) and cadmium(II) removal from aqueous solution using processed walnut shell: kinetic and equilibrium study. Toxicol Environ Chem.

[32] Gautam RK, Mudhoo A, Lofrano G, Chattopadhyaya MC (2014). Biomass-derived biosorbents for metal ions sequestration: adsorbent modification and activation methods and adsorbent regeneration. J Environ Chem Eng.

[33] Mathabatha TIK, Matheri AN, Belaid M (2023). Peanut shell-derived biochar as a low-cost adsorbent to extract cadmium, chromium, lead, copper, and zinc (heavy metals) from wastewater: circular economy approach. CircEconSust.

[34] Mohammadifard A, Allouss D, Vosoughi M, Dargahi A, Moharrami A (2022). Synthesis of magnetic Fe3O4/activated carbon prepared from banana peel (BPAC@Fe3O4) and salvia seed (SSAC@Fe3O4) and applications in the adsorption of Basic Blue 41 textile dye from aqueous solutions. Appl Water Sci.

[35] Li Y, Liu J, Yuan Q, Tang H, Yu F, Lv X (2016). A green adsorbent derived from banana peel for highly effective removal of heavy metal ions from water. RSC Adv.

[36] Dargahi A, Samarghandi MR, Shabanloo A, Mahmoudi MM, Nasab HZ (2023). Statistical modeling of phenolic compounds adsorption onto low-cost adsorbent prepared from aloe vera leaves wastes using CCD-RSM optimization: effect of parameters, isotherm, and kinetic studies. Biomass Conv Bioref.

[37] Chuah TG, Jumasiah A, Azni I, Katayon S, Thomas Choong SY (2005). Rice husk as a potentially low-cost biosorbent for heavy metal and dye removal: an overview. Desalination.

[38] Çelebi H, Gök G, Gök O (2020). Adsorption capability of brewed tea waste in waters containing toxic lead(II), cadmium (II), nickel (II), and zinc(II) heavy metal ions. Sci Rep.

[39] Pinheiro Do Nascimento PF, Lins De Barros Neto E, Fernandes De Sousa J, Trocolli Ribeiro V, De Jesus Nogueira Duarte L, Fonseca Melo RP (2021). Metal ion adsorption using coconut shell powder activated by chemical and physical treatments. Chem Eng & Technol..

[40] Hameed BH (2009). Evaluation of papaya seeds as a novel non-conventional low-cost adsorbent for removal of methylene blue. J Hazard Mater.

[41] Zonato RDO, Estevam BR, Perez ID, Aparecida Dos Santos Ribeiro V, Boina RF (2022). Eggshell as an adsorbent for removing dyes and metallic ions in aqueous solutions. Cleaner Chem Eng..

[42] Huber F, Berwanger J, Polesya S, Mankovsky S, Ebert H, Giessibl FJ (2019). Chemical bond formation showing a transition from physisorption to chemisorption. Science.

[43] Obregón-Valencia D, Sun-Kou MDR (2014). Comparative cadmium adsorption study on activated carbon prepared from aguaje (*Mauritia flexuosa*) and olive fruit stones (*Olea europaea* L.). J Environ Chem Eng.

[44] Shokoohi R, Dargahi A, Azami Gilan R, Zolghadr Nasab H, Zeynalzadeh D, Molla MM (2020). Magnetic multi-walled carbon nanotube as effective adsorbent for ciprofloxacin (CIP) removal from aqueous solutions: isotherm and kinetics studies. Int J Chem Reactor Eng.

[45] Jafari S, Zhao F, Zhao D, Lahtinen M, Bhatnagar A, Sillanpää M (2015). A comparative study for the removal of methylene blue dye by N and S modified TiO2 adsorbents. J Mol Liq.

[46] Dargahi A, Rahimpouran S, Rad HM, Eghlimi E, Zandian H, Hosseinkhani A (2023). Investigation of the link between the type and concentrations of heavy metals and other elements in blood and urinary stones and their association to the environmental factors and dietary pattern. J Trace Elem Med Biol.

[47] Farrokhi M, Naimi- Joubani M, Dargahi A, Poursadeghian M, Jamali HA (2018). Investigating activated sludge microbialpopulation efficiency in heavy metals removalfrom compost leachate. Pol J Environ Stud.

[48] Dargahi A, Gholestanifar H, Darvishi P, Karami A, Hasan SH, Poormohammadi A (2016). An Investigation and comparison of removing heavy metals (lead and chromium) from aqueous solutions using magnesium oxide nanoparticles. Pol J Environ Stud.

[49] Anirudhan TS, Sreekumari SS (2011). Adsorptive removal of heavy metal ions from industrial effluents using activated carbon derived from waste coconut buttons. J Environ Sci.

[50] Filice S, Bongiorno C, Libertino S, Compagnini G, Gradon L, Iannazzo D (2021). Structural characterization and adsorption properties of dunino raw halloysite mineral for dye removal from water. Materials.

[51] Abdulrazak S, Hussaini K, Sani HM (2017). Evaluation of removal efficiency of heavy metals by low-cost activated carbon prepared from African palm fruit. Appl Water Sci.

[52] Mohan B, Kamboj A, Virender, Singh K, Priyanka, Singh G (2023). Metal-organic frameworks (MOFs) materials for pesticides, heavy metals, and drugs removal: Environmental safety. Separat Purif Technol..

[53] Ahmed A, Mohamed F, Elzanaty AM, Abdel-Gawad OF (2021). Synthesis and characterization of diphenylamine grafted onto sodium alginate for metal removal. Int J Biol Macromol.

[54] Bui NT, Kang H, Teat SJ, Su GM, Pao C-W, Liu Y-S (2020). A nature-inspired hydrogen-bonded supramolecular complex for selective copper ion removal from water. Nat Commun.

[55] Xu Z, Wang K, Liu Q, Guo F, Xiong Z, Li Y (2018). A bifunctional adsorbent of silica gel-immobilized Schiff base derivative for simultaneous and selective adsorption of Cu(II) and SO42−. Sep Purif Technol.

[56] Jumina, Priastomo Y, Setiawan HR, Mutmainah, Kurniawan YS, Ohto K (2020). Simultaneous removal of lead(II), chromium(III), and copper(II) heavy metal ions through an adsorption process using C-phenylcalix[4]pyrogallolarene material. J Environ Chem Eng.

[57] Zheng Y, Rao F, Zhang M, Li J, Huang W (2021). Efficient, selective, and reusable metal–organic framework-based adsorbent for the removal of Pb(II) and Cr(VI) heavy-metal pollutants from wastewater. Clean Eng Technol.

[58] Rahaman MH, Md. slam A, Md. slam M, Md. Rahman A, Alam SMN (2021). Biodegradable composite adsorbent of modified cellulose and chitosan to remove heavy metal ions from aqueous solution. Curr Res Green Sustain Chem..

[59] Mahmud M, Md. Mossain S, Bin Mobarak M, Sultana S, Sharmin S, Ahmed S (2022). Co-precipitation synthesis of non-cytotoxic and magnetic cobalt ferrite nanoparticles for purging heavy metal from the aqueous medium: Pb(II) adsorption isotherms and kinetics study. Chem Ecol.

[60] Mohammadi SZ, Mofidinasab N, Karimi MA, Mosazadeh F (2020). Fast and efficient removal of Pb(II) ion and malachite green dye from wastewater by using magnetic activated carbon–cobalt nanoparticles. Water Sci Technol.

[61] Paris EC, Malafatti JOD, Musetti HC, Manzoli A, Zenatti A, Escote MT (2020). Faujasite zeolite decorated with cobalt ferrite nanoparticles for improving removal and reuse in Pb2+ ions adsorption. Chin J Chem Eng.

[62] El Mouden A, El Messaoudi N, El Guerraf A, Bouich A, Mehmeti V, Lacherai A (2023). Multifunctional cobalt oxide nanocomposites for efficient removal of heavy metals from aqueous solutions. Chemosphere.

[63] Anuma S, Mishra P, Bhat BR (2021). Polypyrrole functionalized Cobalt oxide Graphene (COPYGO) nanocomposite for the efficient removal of dyes and heavy metal pollutants from aqueous effluents. J Hazard Mater.

[64] Jayalakshmi R, Jeyanthi J, Aswin Sidhaarth KR (2022). Versatile application of cobalt ferrite nanoparticles for the removal of heavy metals and dyes from aqueous solution. Environ Nanotechnol Monit Manag.

[65] Alves Macedo JC, Sartori Jeunon Gontijo E, Gómez Herrera S, Rangel EC, Komatsu D, Landers R (2021). Organosulphur-modified biochar: an effective green adsorbent for removing metal species in aquatic systems. Surf Interfaces.

[66] Srivastava V, Kohout T, Sillanpää M (2016). Potential of cobalt ferrite nanoparticles (CoFe2O4) for remediation of hexavalent chromium from synthetic and printing press wastewater. J Environ Chem Eng.

[67] Holzwarth U, Gibson N (2011). The Scherrer equation versus the “Debye-Scherrer equation”. Nat Nanotech.

[68] Li A, Deng H, Jiang Y, Ye C (2020). High-efficiency removal of Cr(VI) from wastewater by Mg-loaded biochars: adsorption process and removal mechanism. Materials.

[69] Bayuo J (2021). Decontamination of cadmium(II) from synthetic wastewater onto shea fruit shell biomass. Appl Water Sci.

[70] Wang C, Boithias L, Ning Z, Han Y, Sauvage S, Sánchez-Pérez J-M (2017). Comparison of Langmuir and Freundlich adsorption equations within the SWAT-K model for assessing potassium environmental losses at basin scale. Agric Water Manag.

[71] Ghoneim MM, El-Desoky HS, El-Moselhy KM, Amer A, Abou El-Naga EH, Mohamedein LI (2014). Removal of cadmium from aqueous solution using marine green algae, *Ulva lactuca*. Egypt J Aquat Res.

[72] Farooq U, Kozinski JA, Khan MA, Athar M (2010). Biosorption of heavy metal ions using wheat based biosorbents – a review of the recent literature. Biores Technol.

[73] Giraldo L, Moreno-Piraján JC (2008). Pb2+ adsorption from aqueous solutions on activated carbons obtained from lignocellulosic residues. Braz J Chem Eng.

[74] Haso HW, Dubale AA, Chimdesa MA, Atlabachew M (2022). High performance copper based metal organic framework for removal of heavy metals from wastewater. Front Mater.

[75] Meena AK, Kadirvelu K, Mishraa GK, Rajagopal C, Nagar PN (2008). Adsorption of Pb(II) and Cd(II) metal ions from aqueous solutions by mustard husk. J Hazard Mater.

[76] Kowsura C, Pangkumhang B, Jutaporn P, Tanboonchuy V, Department of Environmental Engineering, Faculty of Engineering, Khon Kaen University (2017). Isotherm models of heavy metal sorption onto zinc-tricarboxylic. IJCEA..

[77] Gupta NK, Kim S, Bae J, Kim KS (2021). Chemisorption of hydrogen sulfide over copper-based metal–organic frameworks: methanol and UV-assisted regeneration. RSC Adv.

[78] Almasi A (2016). Efficiency of a constructed wetland in controlling organic pollutants, nitrogen, and heavy metals from sewage. J Chem Pharm Sci.

[79] Alavi N, Shamshiri S, Pariz Z, Dargahi A (2016). Evaluating the palm leaves efficiency as a natural adsorbent for removing cadmium from aqueous solutions: isotherm adsorption study. J Pharm Technol.

[80] Gnanasekaran G, Arthanareeswaran G, Mok YS (2021). A high-flux metal-organic framework membrane (PSF/MIL-100 (Fe)) for the removal of microplastics adsorbing dye contaminants from textile wastewater. Separat Purif Technol..

[81] Liu S (2015). Cooperative adsorption on solid surfaces. J Colloid Interface Sci.

[82] Murphy OP, Vashishtha M, Palanisamy P, Kumar KV (2023). A review on the adsorption isotherms and design calculations for the optimization of adsorbent mass and contact time. ACS Omega.

[83] Tseng R-L, Wu F-C (2008). Inferring the favorable adsorption level and the concurrent multi-stage process with the Freundlich constant. J Hazard Mater.

