# Correction to: Adsorptive removal of heavy metals from wastewater using Cobalt-diphenylamine (Co-DPA) complex

**DOI:** 10.1186/s13065-024-01216-0

**Published:** 2024-06-14

**Authors:** Mesfin Yimer, Shagufi Naz Ansari, Biniyam Abdu Berehe, Krishna Kanthi Gudimella, Gangaraju Gedda, Wubshet Mekonnen Girma, Nazim Hasan, Shadma Tasneem

**Affiliations:** 1 Department of Chemistry, College of Natural Science, Wollo University, P.O. Box:1145, Dessie, Ethiopia; 2 Department of Chemistry, School of Engineering, Presidency University, Bangalore, Karnataka 560064 India; 3 Department of Chemistry, School of Science, GITAM (Deemed to Be University), Rudraram, Telangana 502329 India; 4 Central Research Laboratory, K S Hegde MedicalAcademy, NITTE (Deemed to Be University), Deralakatte, Mangaluru, Karnataka 575018 India; 5 Department of Animal Science & Technology and BET Research Institute, Chung-Ang University, Anseong, Gyeonggi-do 17546 Republic of Korea; 6 Department of Chemistry, College of Science, Jazan University, P.O. Box 114, Jazan, 45142 Kingdom of Saudi Arabia

**Correction to: BMC Chemistry (2024) 18:23**

10.1186/s13065-024-01128-z

Following publication of the original article [1], the authors noticed Co-DPA mislabeled as Cu-DPA in “Keywords” and “Recovery studies” section. Although the error was present, these corrections do not affect the interpretation and conclusions of the original article as a whole. The authors apologize for any inconvenience this may have caused the readers.

**Keywords**

Wastewater, Adsorption, Co-DPA, Real sample, Freundlich isotherm

***Recovery studies***.

Reusing the adsorbent by regenerating its adsorption characteristics is an economic necessity in many applications. With growing raw material and wastewater treatment process expenses, the allure of product recovery technologies has grown dramatically [74, 77]. The Co-DPA adsorbent was evaluated for its reusability. The recycling results shown in Fig. 7 show that it maintains its activity despite a decline in metal ion removal efficiency, and it is pH dependent and performed by adding HCl and NaOH to solution.

In a typical experiment, 0.01 M HNO_3_ or HCl and 0.005 M NaOH eluents were added to the solution to perform the recycling test. Metals were initially adsorbed on Co-DPA from 60 mL solutions containing 80 mg/L metal ions at pH 3 (Cr) and 7 (Cd &Pb). The Co-DPA were then stripped with 30 mL eluent while agitating at 25 °C for 30 min. The Co-DPA complex was separated, and the metal ion concentration in the supernatant was determined. The adsorption–desorption cycles were done three times for each measurement.
